# Eco-Friendly Fired Brick Produced from Industrial Ash and Natural Clay: A Study of Waste Reuse

**DOI:** 10.3390/ma14040877

**Published:** 2021-02-12

**Authors:** Neslihan Doğan-Sağlamtimur, Ahmet Bilgil, Magdalena Szechyńska-Hebda, Sławomir Parzych, Marek Hebda

**Affiliations:** 1 Department of Environmental Engineering, Niğde Ömer Halisdemir University, 51240 Niğde, Turkey; 2 Department of Civil Engineering, Niğde Ömer Halisdemir University, 51240 Niğde, Turkey; abilgil@ohu.edu.tr; 3 The *Franciszek Górski* Institute of Plant Physiology Polish Academy of Sciences, Niezapominajek 21, 30-239 Cracow, Poland; szechynska@wp.pl; 4 The Plant Breeding and Acclimatization Institute—National Research Institute, Radzików, 05-870 Błonie, Poland; 5 Institute of Materials Engineering, Faculty of Material Engineering and Physics, Cracow University of Technology, Warszawska 24, 31-155 Cracow, Poland; slawomir.parzych@pk.edu.pl (S.P.); mhebda@pk.edu.pl (M.H.)

**Keywords:** bottom ash, clay, environment, fired brick, reuse, waste

## Abstract

Bottom ash (BA) is an industrial solid waste formed by the burning of coal. The environmental problems and storage costs caused by this waste increase with every passing day. In this study, the use of BA as an additive (clay substitute) in fired brick production was investigated. The study consisted of two stages. In the first stage, cylinder blocks were produced from clay used in brick production. The second stage was the examination of the experimental substitution of clay with 10, 20, 30 and 40% BA. Samples were fired at 900, 1000, 1100 and 1150 °C to produce fired brick samples. The unit weight, compressive strength (before and after freeze–thawing) and water absorption were analyzed for the samples. The unit weight values decreased in the samples containing BA. The mechanical properties met the conditions prescribed in the relevant standards; i.e., all of the samples fired at 1100 and 1150 °C had a sufficient compressive strength over 20 MPa. The high potential of fired bricks for the construction industry was proved. BA can be used as a clay substitute, while the developed protocol can be used to effectively produce fired bricks.

## 1. Introduction

Today, many countries are in the process of rapid industrial development. In spite of the benefits of industrial development, some negative effects are a fact that certainly cannot be ignored. Millions of tons of residual waste, and that produced each year, contributes substantially to environmental disasters. Among other factors, rapid growth in construction activities increases construction waste problems around the world. To reduce these negative impacts, a comprehensive understanding of construction waste generation and management is needed. On the other hand, the construction industry can consume waste in very high volumes. The evaluation of these wastes as construction materials, preventing an increase in waste stocks, is the subject of many scientific studies [1,2,3,4,5].

As in many countries, coal is preferred as a source of energy for the industrial development of Turkey. One of the negative consequences of coal usage for humanity and for the environment is the waste ash problem. Generally, Turkey has low-calorie coal deposits and excessive bottom ash (BA) resulting from its use. Very little of this huge amount of BA is used in the production of cement and concrete [6]. This waste cannot be managed appropriately. Reusing this waste ash through beneficial recovery mechanisms can be of importance in terms of both environmental protection and economic gains. As the coal ash has an organic structure, it can be used in the production of clay bricks.

In many studies focused on fired clay bricks, it has been reported that there is a strong relationship between density, porosity and thermal conductivity. Clay brick, when substituted with an organic substance of very low heat conductivity, has a higher porosity and reduced density. These properties vary widely depending on the production conditions, including the drying and firing temperatures, firing time or type of oven used for firing. The particle size also has a considerable effect on the thermal conductivity. During firing, the added matter is consumed, leaving voids that increase the porosity [7].

Suitable physical and mechanical properties as well as good insulation behavior are required for bricks. Fired clay bricks are mostly used to form enclosures; therefore, their properties should ensure good insulation. Buildings’ thermal energy, required for the heating and air conditioning of buildings, accounts for approximately 40% of the overall energy consumed in the world. This represents 36% of the global CO_2_ emissions [8,9], and previous studies estimate that 50% of this energy is lost through walls [9,10].

Çiçek and Tanrıverdi [11] investigated the possibilities of BA for its use in low- and high-thermal-insulation bricks. The researchers tested brick samples produced using the curing method for mixtures of fly ash (FA), sand and hydrated lime with steam at high pressures, and obtained positive results. Kızgıt et al. [12] investigated the possible usage of FA in the Çatalağzı Thermal Plant (Turkey) for fired brick production and showed that bricks of appropriate quality can be produced by mixing 30 to 40% FA with brick material. Similarly, Bai et al. [13] found that concretes with the natural sand replaced with 30% BA had compressive strengths ranging from 40 to 60 N/mm^2^ without the drying shrinkage properties of the concrete being detrimentally affected. They also reported that the processability, carbonation and water absorption percentage were increased, whereas the chloride permeability was decreased. Yüksel et al. [14] investigated the possible usage of BA as an aggregate in low-density briquette production. The positive results of the tests enabled directly applying the briquettes in the construction industry. Bentli et al. [15] added 2.5%, 5%, 10% and 15% FA to brick paste and found that this additive increased the unit weight, and caused no significant changes in the drying, firing and total shrinkage, while decreasing the water absorption and compressive strength of fired bricks. By contrast, Linling et al. [16] found that FA added to brick paste as an alternative to clay increased the compressive strength value, decreased the water absorption, eliminated the cracking, and increased the freeze–thaw resistance for the bricks fired at 1050 °C. Topçu and Işıkdağ [17] added perlite to bricks produced from clay in different ratios. The bricks containing 24% perlite showed the best unit weight and heat permeability, bricks containing 30% perlite showed the best compressive strength, and bricks containing 31% perlite exhibited the best shrinkage. Demir [18] added inflammable organic materials including sawmill powder, tobacco pulp and grass to clay in 0%, 2.5%, 5% and 10% ratios by weight. It was found that organic material has significant effects on the mechanical properties and porosity formation in the clay structure.

Scientists have used different methods [19] to increase the porosity in modern brick production. A wide variety of waste materials have also been tested as sources of additives, including paper production residue [20]; cigarette butts [21]; rice husk ash [22,23]; kraft pulp production residue [24]; waste tea [25]; sawdust [26]; vine shoots [27]; vegetable matter [28]; pineapple leaf fibers [29]; organic matter [7]; sugarcane bagasse ash waste [30]; incorporated biomasses [31]; corn cobs [32]; organic and inorganic wastes [33]; ice husks, sawdust, coir pith and fly ash [16,34,35,36,37,38]; granite sawing wastes [39]; municipal solid waste incinerator slag [40]; kaolin fine quarry residue, granulated blast-furnace slag and granite–basalt fine quarry residue [41]; Waelz slag and waste foundry sand [42]; industrial nanocrystalline aluminum sludge [43]; waste glass [44]; construction and demolition waste [45]; and crumb rubber, cement kiln dust, mine tailings, slags, wood sawdust, cotton waste, limestone powder and petroleum effluent treatment plant sludge [19,46,47,48].

The methods used to make bricks from clay include molding, dry pressing and extrusion. Once the bricks take their form, they are dried and fired in a kiln. Properly fired clay bricks have many desirable qualities, including high compressive strength, a porosity that allows them to absorb and release moisture, fire resistance, insulative properties regarding both heat and sound, and durability under a wide range of temperatures and weather conditions. The desirable characteristics of bricks include uniformity in color, size and shape, and they should be free from cracks and flaws. In addition, the compressive strength of bricks should be suitable for their intended use [49].

Although many studies have been conducted, the commercial production of bricks from waste materials is still very limited [50]. This study concerns eco-friendly fired brick production by applying BA from the international Göknur Foodstuff Co. Niğde Factory (Niğde, Turkey) (BA_GFCNF_) and clay from the Kolsuz Region at Niğde Province (C_NKR_). BA_GFCNF_, unused or discarded, can cause environmental pollution; however, we proved that it can be applied as an additive to produce lightweight bricks used in construction technology. Using waste ash resolves the environmental problems, allows manufacturers to replace raw material inputs from natural resources with reused materials, and reduces resource depletion, thus contributing to circular economy, zero waste, green engineering and sustainable development approaches.

## 2. Materials and Methods

### 2.1. Materials

The raw materials used for the study were BA_GFCNF_ and C_NKR_. Approximately 50 tons/day (17,000–19,000 tons/year) of BA_GFCNF_ is generated as a result of burning coal for energy purposes and sent to the Landfill of Niğde Municipality (Turkey) from the factory. The field area with clay is located in the northwest of NKR on the Niğde-Adana highway running for 40 km across Niğde Province. For the study, BA_GFCNF_ was supplied by the factory, while C_NKR_ was collected from different points of the field in pellet form with various sizes of particles. The BA_GFCNF_ and C_NKR_ were brought to the Waste Technologies Laboratory of Niğde Ömer Halisdemir University, and samples were prepared. Then, chemical and physical analyses were carried out in the materials laboratory of the ÇİMSA Cement Factory Inc. in Niğde, Turkey. The results of the chemical analysis determined by Panalytical/Zetium XRF (Malvern Panalytical, Malvern, UK) performed for the raw materials (in oxides wt%; experimental error, ±0.05 wt%) are given in Table 1. The amounts of CaO, SO_3_, Al_2_O_3_ and Fe_2_O_3_ were significantly higher (>400, 80, 9 and 5%, respectively) in BA_GFCNF_ than in C_NKR_, and the other components were much lower (<9%), respectively. The other components, SiO_2_, MgO, and K_2_O, were significantly lower (<55, 20 and 20%, respectively).

The compact unit weight of the BA_GFCNF_ was γ = 0.89 g cm^−3^. The amount of flammable material in this ash was 43% after heat treatment at 800 °C for two hours. C_NKR_ pellets were sieved through a 500 µm square-mesh sieve to remove sand and pebbles from the ground samples. The compact unit weight (UW) value of the C_NKR_ was found to be γ = 1.67 g cm^−3^; the percentage of flammable material was 0.5%.

The fired bricks produced in this study were 190 × 90 × 50 mm^3^ in size. The weight of an individual brick having the maximum UW (FB_0–900_) was 1.62 kg. Therefore, the weight of all the samples in this study was under 3 kg.

**Figure 1:**
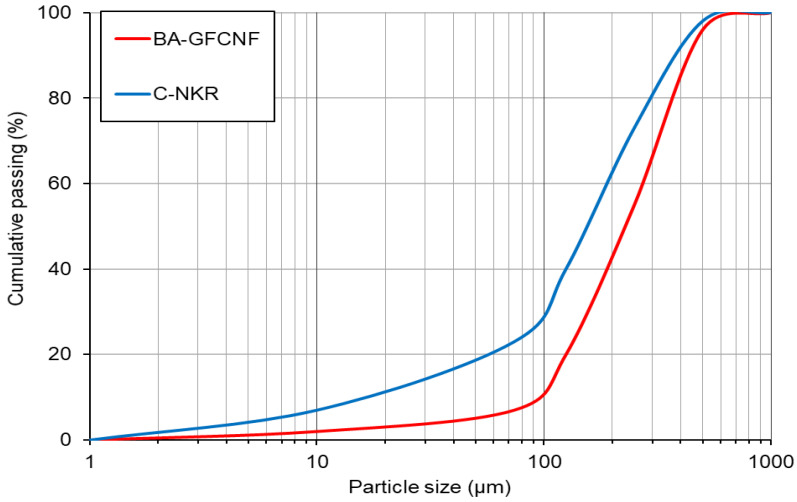
Particle size distributions of the BA_GFCNF_ and C_NKR_.

A sieve analysis (UTEST UGT0411, Ankara, Turkey) was used to determine the particle size distributions of the BA_GFCNF_ and C_NKR_ (Figure 1). The median diameter (d_50_), which is the value of the particle diameter at 50% in the cumulative distribution, was approximately 225 and 155 μm for BA_GFCNF_ and C_NKR_, respectively. The range of the apparent particle density on an oven-dried basis of BA_GFCNF_ was 1.9–2.1 g cm^−3^, and that of C_NKR_ was 1.8–2.5 g cm^−3^.

### 2.2. Methods

The study was conducted in two stages. In the first stage, C_NKR_ (0% BA_GFCNF_) was used to produce the fired brick control samples. These samples were used for the comparative analysis during the second stage of the study, which comprised the production of C_NKR_ and BA_GFCNF_ mixtures in the ratios 1:10, 2:8, 3:7 and 4:6.

**Figure 2:**
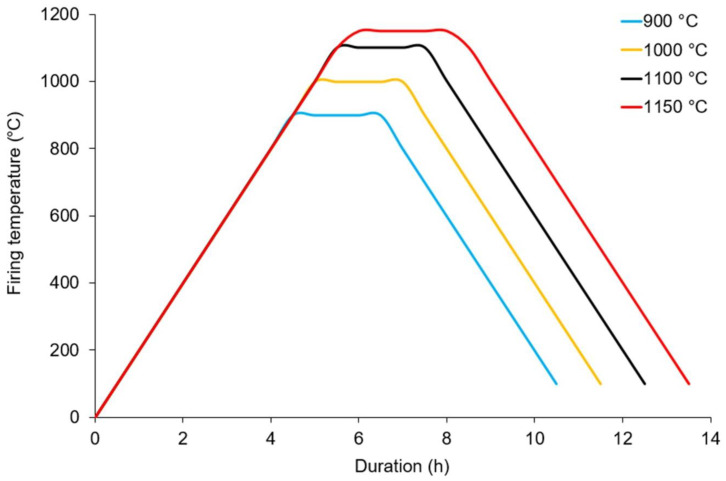
Temperature profiles used for brick firing.

The protocol for fired brick production included the following steps: (1) BA_GFCNF_ and C_NKR_ were oven dried, and a visual examination was applied to determine the consistencies of all the mixtures; (2) the UW values were determined for the mixtures; (3) the stirring of the mixtures for 3 min at low revolutions was carried out using a table type mixer (UTEST UTG-0130, Ankara, Turkey); (4) tap water was added, and the mixtures were stirred to a moist consistency for the next 3 min; (5) the one-step filling of a cylindrically shaped mold (φ = 7 cm and h = 12 cm) with the mixtures was performed with care in order to prevent layer and crack formation; (6) a pressure of about 2 kg cm^−2^ was applied with a laboratory press (UTEST UTC-5700, Ankara, Turkey); (7) the samples were dried in the oven at 105 ± 5 °C for 24 h; (8) the samples were fired at 900, 1000, 1100 and 1150 °C for 2 h (with a 2.5 °C/min heating/cooling rate) in a laboratory oven (Kaleo RS150, Kütahya, Turkey). The temperature profiles used for the brick firing are shown in Figure 2.

Forty-five samples were produced for each type of firing (a total of 180 samples). In each group, 3 samples were used for freeze–thaw resistance tests, and 3 samples were used for the determination of physical and mechanical properties. One set of samples served as a control.

The properties of the fired bricks were tested using Turkish standards and European norms (TS EN): the unit weight (UW) with TS EN 772-13; water absorption (WA) with TS EN 772-11; compressive strength (CS) with TS EN 772-1 + A1; freeze–thaw resistance with TS EN 772-18 [51,52,53,54,55]. The porosity or void fraction was a measure of the “empty” spaces in a material and is expressed as a fraction of the volume of voids over the total volume. The WA of the samples by volume were calculated, which also represent the apparent porosity [56].

For the calculation of the firing shrinkage (FS), the following equation was used:

S_f_ = W_f_ − W_od_/W_od_ × 100 (%) (1)

where W_f_ is the sample weight after firing, and W_d_ is the sample weight after oven drying.

For the calculation of the UW, the following equation was used:

(2) $$\gamma =\frac{m_{\mathrm{ad}}}{V_{g}}\;(g/{\mathrm{cm}}^{3})$$

where m_ad_ is the air-dried mass, and V_g_ is the gross volume of the samples.

Using the data obtained, the WA values of the samples by volume (A_w_) were calculated:

(3) $$\mathrm{Aw}=\frac{m_{\mathrm{sa}}-m_{d}}{m_{\mathrm{sa}}-m_{s}}100\;(\%)$$

where m_sa_ is the saturated mass, m_d_ is the dried mass, and m_s_ is the sample mass (according to Archimedes’ principle, water scale).

For the calculation of the CS, the following equation was used:

(4) $$\sigma =\frac{F}{A}(\mathrm{MPa})$$

where F is the load applied, and A is the area.

For the calculation of the compressive strength after freezing (CS-AF), the following equation was used:

(5) $$\Delta f=\frac{f_{s}-f_{\mathrm{af}}}{f_{s}}100\;(\%\;\mathrm{MPa})$$

where f_s_ is the CS of the samples, and f_af_ is the samples’ CS-AF.

## 3. Results and Discussion

Major components of clay minerals are aluminum, silicon and oxygen. As their ratio changes in different types of clays, the firing time and temperature should be optimized each time. Similarly, high-alumina-containing ash is a good candidate from waste materials for synthesizing mullite ceramics; thus, it can be used to fabricate insulation refractories and ceramic tiles. Silicon carbide is an encouraging non-oxide ceramic, and silica-rich wastes are favorably used in order to obtain glass or glass ceramic [57,58]. In our studies, we compared brick samples prepared with different compositions of clay (50.97% SiO_2_; 11.58% Al_2_O_3_) vs. BA (12.68% SiO_2_; 27.36% Al_2_O_3_) and fired at various temperatures. The feasibility of using a high amount of BA, even up to 60 wt%, as an alternative raw material in clay-based ceramic compositions and their higher strength and density values with increasing temperature were shown [59]. For our study, we chose contents of BA addition in the range 0 to 40%, and the temperature ranged from 900 to 1150 °C.

Under visual examination, deformation, cracks or color changes were not observed in the samples fired at 900, 1000 and 1100 °C. However, color changes developed in the samples fired at 1150 °C. Usually, brick achieves its color through the minerals in the fired clay. This provides a durable color that never fades or diminishes. The color change suggests that the firing temperature of 1150 °C was too high. It caused phase changes during the firing process, and thus, the temperature was not suitable for this type of raw material. Probably, metallic oxides, which act as fluxes promoting the fusion of the particles at lower temperatures (particularly those of iron, magnesium and calcium), can influence the color of bricks fired at temperatures exceeding the optimum. One can consider the cooling time as a factor that can influence changes in color development. After the firing temperature had peaked and was maintained for a prescribed time, the cooling process began, an important stage in brick manufacturing. The rate of cooling has a direct effect on color. However, the cooling time and its kinetics were the same during our experiment, supporting our statement and suggesting that the temperature optimum should be carefully chosen to help control color during manufacturing a certain brick body.

**Figure 3:**
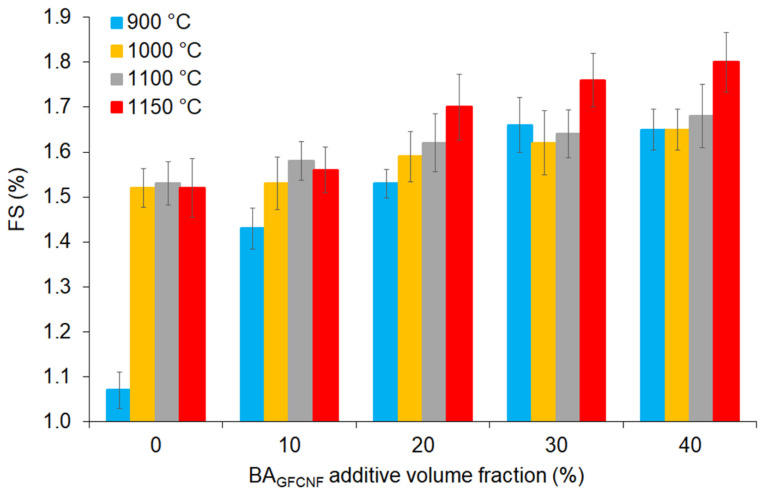
The values of FS depending on the BA_GFCNF_ content, which ranged from 0 to 40%, and firing temperatures applied in the range of 900 to 1150 °C.

**Figure 4:**
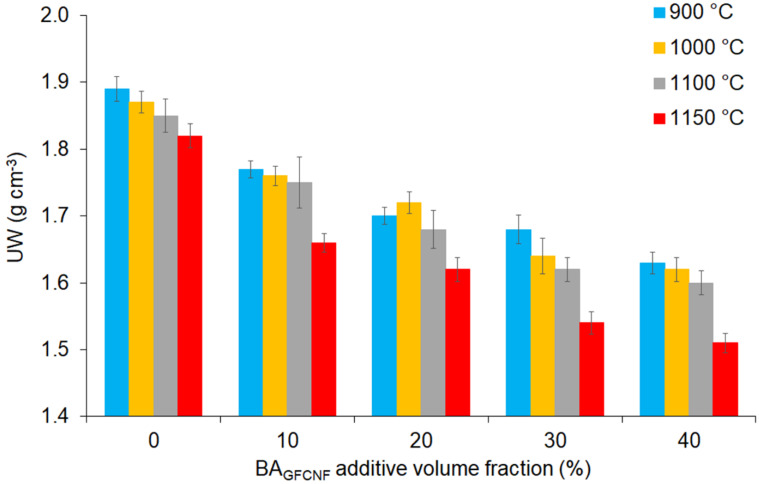
The values of UW depending on the BA_GFCNF_ content, which ranged from 0 to 40%, and firing temperatures applied in the range of 900 to 1150 °C.

**Figure 5:**
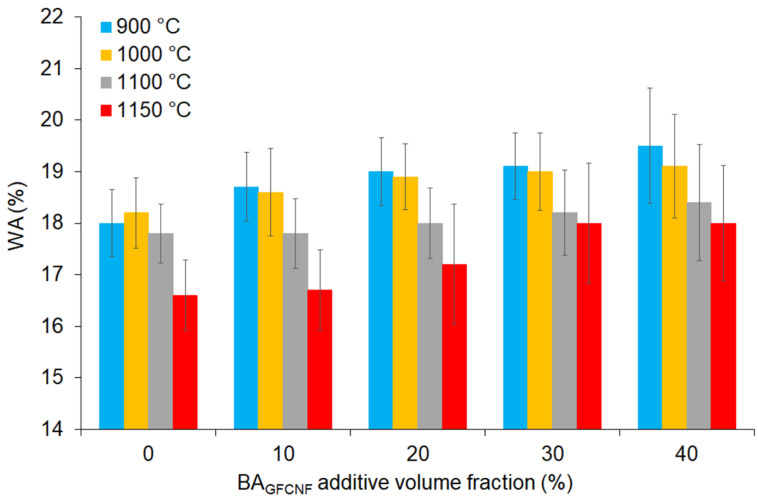
The values of WA depending on the BA_GFCNF_ content, which ranged from 0 to 40%, and firing temperatures applied in the range of 900 to 1150 °C.

**Figure 6:**
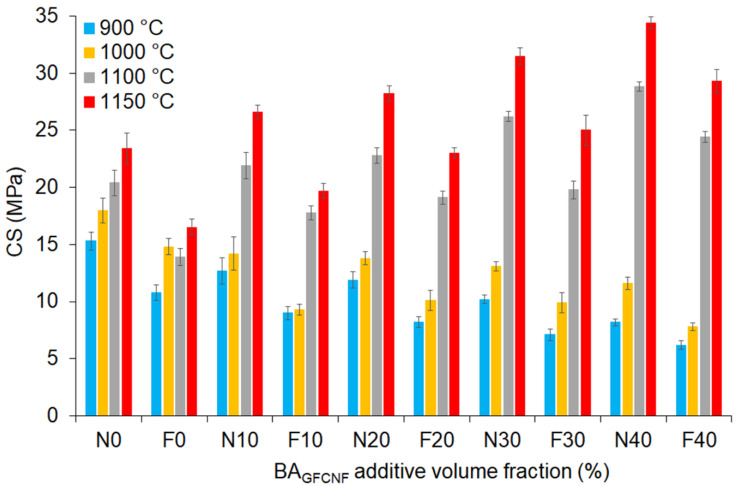
The values of CS (N) and CS-AF (F) of bricks after different temperature treatments (900–1150 °C). For N and F, numbers indicate BA_GFCNF_ content.

The collective results of the tests applied on the fired bricks during the production process in this study are given in Table 2 and subsequent Figure 3, Figure 4, Figure 5 and Figure 6. Table 2 also presents the statistics of the standard error and the significance determined by Student’s t-test. In most cases, for the UW, CS and CS-AF but not for the FS, WA and *p*, low *p* values (denoted by one, two or three asterisks) were obtained. This confirmed significant differences from FB0 within each set of samples produced from C_NKR_ and BA_GFCNF_ mixtures at the different ratios and fired at the same temperature as well as from FB900 within each set of samples produced from a C_NKR_ and BA_GFCNF_ mixture at the same ratio and fired at different temperatures. Therefore, different methods of fired brick production that include different composition or temperature factors (particularly the highest ones) provide divergent analytical results and final product properties.

The quality of the brick could be measured by examining the shrinkage of the samples. The shrinkage in the ceramic process is a significant parameter, since structural change and solidification, implying densification, may create tensions and failures in fired bricks [59]. All the FS values obtained for the fired bricks (Table 2 and Figure 3) were lower than 2%. The progressive addition of BA_GFCNF_ to the C_NKR_ causes an increase in the recorded shrinkage value, and the highest BA_GFCNF_ volume fraction (30–40%) had the most significant influence on the parameter changes. This effect was similar at each of the applied firing temperatures; however, the higher the temperature, the higher the FS that was observed. The effect was mainly dependent on the combustion of carbon in the ash fraction and organic material in the clay fraction at elevated temperatures. Starting from 1100 °C, liquid phase sintering also becomes a very important mechanism [60]. It was emphasized that liquid phase sintering was existent if there was a liquid phase that coexists with particulate solids during the sintering process. The liquid phase can fill some internal pores by a diffusion mechanism, particularly if organic combustion changes the internal structure and forces. In the case of a solid state, the sintering process develops a new atomic bonding between particles, followed by grain growth, which creates a strong structure with significant shrinkage.

Figure 4 shows the UW values of the fired bricks as a function of the BA_GFCNF_ content in the range of 0–40% and firing temperatures in the range of 900 to 1150 °C. The UW of the samples decreased significantly as the ratio of BA_GFCNF_ to C_NKR_ increased. It was found that the firing temperatures ranging from 900 to 1100 °C affected the UW slightly (in most cases, the differences were insignificant) in the sample groups having the same component ratio. However, the UW values significantly decreased in the samples fired at 1150 °C for all the mixture types (Table 2). This difference in the sintering behavior was related to the presence of a 43% residual carbon phase, as was shown after heat treatment at 800 °C for two hours. Further heat treatment led to the burning of this residual phase and was directly associated with an increase in porosity. An increased volume of voids formed during the combustion of carbon in the ash fraction and organic material in the clay fraction at elevated temperatures was also reported earlier [59]. The increased volume of voids can also result in improved thermal isolation properties. Good bricks should have low thermal conductivity so that houses keep cool in summer and warm in winter. Indeed, the thermal conductivity of the fired brick in our study was low (0.19 W m^−1^ K^−1^). Therefore, the produced fired bricks could create a zone of thermal comfort within buildings.

Indeed, the WA provides more information for improved open porosity. It is believed that low values imply good resistance to the natural environment and the acceptable permeability of bricks [59]. The relationships between the WA, BA_GFCNF_ content and firing temperatures are given in Figure 5. Although changes in the BA_GFCNF_ content and elevated temperatures affected the WA slightly, the WA decreased as the firing temperatures increased for each type of BA_GFCNF_ and C_NKR_ mixture. The most remarkable changes (statistically significant) were found at temperatures above 1100 °C. Considering the increase in shrinkage along with a temperature increase, the WA decrease confirmed that the liquid phase filled pores, but only in part, and the relatively light weight and structure of the bricks were maintained. It was related to the phase changes in the material. Generally, a clay-based material mixed with water (to make it soft and flexible) and other materials, squashed into shape and then fired at high temperature in a kiln (above 900 °C) turns into a compound called mullite. SiO_2_ released according to the equation 3(Al_2_O_3_.2SiO_2_)→3Al_2_O_3_.2SiO_2_ + SiO_2_ forms a glass phase with closed internal pores. This structure, related to ceramic [61], is waterproof. The voids surrounded with a ceramic structure prevented water penetration into the internal structure, and thus, water adsorption/desorption was inhibited in our samples. Indeed, the open porosity values were complementary to the WA results (Table 2). Similarly, the fired bricks with a high-volume ratio of FA presented a high CS and a low WA capacity, and with an increase in the firing temperature, the CS increased, and the WA decreased [34]. Therefore, the increase in the porosity can be controlled by the addition of BA_GFCNF_ and temperature of firing.

Depending on the country, there are different standards specifying brick grades according to the CS values. For instance, withdrawn European standards specified a minimum strength of 5 MPa for burnt clay bricks (BS 3921, 1985; TS 705, 1985). Figure 6 shows that the CS values of the brick samples prepared with different contents of BA_GFCNF_ additive ranged from 10 up to 40%. It has been observed that along with an increase in BA_GFCNF_ content in the samples, the CS values decreased for the samples fired at 900 and 1000 °C. On the other hand, the samples with increased BA fractions in the mixtures (BA_GFCNF-30_ and BA_GFCNF-40_) showed a linear increase in CS values in comparison to the control sample, provided that the firing temperature was 1100 or 1150 °C. The differences were statistically significant, in both cases, for the BA_GFCNF_ content and firing temperature (Table 2). This effect was associated with the formation of a ceramic structure, the reactions between BA_GFCNF_ and C_NKR_, and the formation of silicon carbide at temperatures above 1000 °C.

Figure 6 also presents the results of the freeze–thaw test, which was applied to determine the resistance of the samples to external factors. It was shown that the freeze–thaw test did not correlate the strength of the bricks with their frost resistance or the forms of damage [62]. By contrast, we found that similar to CS, the average CS-FA values were reduced after the freeze–thaw tests to 27, 26, 25 and 22% for the samples with 10, 20, 30 and 40% BA_GFCNF_, respectively. Moreover, the samples fired at higher temperatures were less affected by the freeze–thaw test. This effect could be related to reduced porosity. Indeed, it has been shown that bricks become damaged after freeze–thaw tests in a specific way depending on their structure of porosity and the spatial arrangement of the texture components [62]. The freeze–thawing induces the effects of ice crystallization inside the porous system. Three forms of frost damage influence the final brick properties, namely, powdering, flaking and cracking. Bricks with relatively high shares of pores with diameters of 1–10 µm in the total population of pores undergo these types of frost damage; i.e., they are characterized by a lack of frost resistance [62]. A continuous reduction in porosity and a significant increase in the pore fraction with a radius >1 mm occurred as the firing temperature rose and smaller pores coalesced. On the other hand, as the BA_GFCNF_ content and firing temperatures increased, the samples tended to form silicon carbide and a ceramic structure, which caused a higher resistance to the freezing temperatures. Considering the CS values, it was proved that the fired bricks comply with the standards, and they can be used as wall elements. In fact, according to ASTM C67 (1992) [63], the minimum required CS for a paving brick subjected to light traffic is 17.2–20.7 MPa. ASTM C62 (2005) [64] for building and facing bricks, respectively, correlate the CS with the weathering resistance, specifying a minimum value of 20.7 MPa, for the bricks to not be susceptible to degradation. In these terms, all the samples, regardless of the compositions, fired at 1100 and 1150 °C should be durable against severe weathering. Moreover, the FB_40–1100_, FB_20–1150_, FB_30–1150_ and FB_40–1150_, samples also met these requirements after the freezing measurements.

The optimized protocol for producing fired bricks with the addition of BA and their improved properties enable using them in construction technology. In Turkey, bricks are covered (plastered) and do not come into direct contact with water; thus, no efflorescence test was performed. Therefore, the TS EN 772-5 standard (Methods of test for masonry units—Part 5: Determination of the active soluble salts content of clay masonry units) was withdrawn as a standard. However, the improved properties of the fired bricks produced during our studies include lower wettability (water impermeability), indicating possibilities of the extended or alternative application of lightweight structural elements. Additionally, for the further reduction of the WA of the fired bricks, applying resin or polymer material on their surfaces can be considered. It is worth mentioning that fired brick production was also tried at 800 °C; however, in the freeze–thaw test, the samples crumbled. Therefore, production without firing from our waste BA and clay is not suitable. Higher temperatures did not improve the properties of the fired brick. Therefore, the applied temperature range was selected correctly. Further experiments can be considered to allow optimizing the protocol steps in detail.

## 4. Conclusions

Turkey has low-calorie coal deposits and excessive BA resulting from its use. We address the reusing of this waste through beneficial recovery mechanisms. The manuscript reports successful fired brick production from C_NKR_ substituted with BA_GFCNF_ (0, 10, 20, 30 and 40%) by using thermal processes (900, 1000, 1100 and 1150 °C). The protocol for fired brick production should include the following steps: the oven drying of BA_GFCNF_ and C_NKR_; the stirring of the mixtures with tap water; the one-step filling of a mold; pressing; oven drying for 24 h; firing for 2 h. The UW of the fired brick samples decreased (1.89 g to 1.51 g cm^−3^), while the porosity and WA (16.6 to 19.5%) increased, along with a linear increase in the BA_GFCNF_ volume fraction. This was a result of the complete burning of coal in the BA_GFCNF_ at higher temperatures, as well as reaching the sintering point and developing a glassy structure in the clay fraction. Firing at temperatures of 1000 °C or above has a positive influence on the microstructure of the brick, promoting a dense structure with low permeability. At these temperatures, the chemical reactions for BA_GFCNF_ led to carbon and silica transformation into silicon carbide. The findings indicate that the physical and mechanical properties of bricks can be controlled to a significant extent by varying the firing temperature and composition of the raw materials. As a result, the present study revealed the potential of producing fired brick from BA_GFCNF_ and C_NKR_ for structural applications. An innovation is the use of a combination of a high content of BA_GFCNF_ and high firing temperature. In the case of BA_GFCNF_, a content of 40% can be recommended and used as a filler and additive material for the clay in fired brick production, and this mixture has a good potential for reusing the waste. For these types of mixture, a temperature of 1150 °C is recommended as the optimal firing procedure. However, some limitations in the use of the procedure may result from the costs, which are an important factor for large-scale production. Due to economic reasons, the firing temperature can be reduced to between 1000 and 1100 °C. The fired bricks can still achieve significantly higher (acceptable) strength. Other benefits of industrial waste ash addition in bricks and other materials that are most often mentioned in the literature [65,66] are (i) the conservation of natural resources, e.g., by replacing natural clay with waste, and (ii) solving disposal problems and protecting the environment. Therefore, fired brick production with BA_GFCNF_ incorporation could be a substantial step towards a decrease in pollution and environmental impact and a good example for industrial symbiosis. This approach will also contribute to green engineering, zero waste, sustainable development and circular economy principles.

## Figures and Tables

**Table 1:** Chemical compositions of the raw materials used in this study.

Compounds	Amount (%)	
	BA_GFCNF_	C_NKR_
SiO_2_	27.36	50.97
Al_2_O_3_	12.68	11.58
Fe_2_O_3_	8.23	7.77
CaO	39.31	9.40
MgO	0.74	3.90
Na_2_O	1.88	1.83
K_2_O	0.43	2.31
SO_3_	5.94	3.13
Others	3.43	9.11

**Table 2:** Physical and mechanical properties of the fired brick samples produced from BA_GFCNF_ and C_NKR_ mixture. Subscripts in the sample descriptions indicate BA_GFCNF_ volume fraction (0–40%) and firing temperature (900–1150 °C), respectively. FS, firing shrinkage; UW, unit weight; WA, water absorption; *p*, porosity; CS, compressive strength; CS-AF, compressive strength after freezing. The results are the means of three measurements; data are presented as mean ± standard error; blue asterisks represent significant differences from FB0 within each set of samples produced from C_NKR_ and BA_GFCNF_ mixtures at the different ratios and fired at the same temperature; red asterisks represent significant differences from FB900 within each set of samples produced from C_NKR_ and BA_GFCNF_ mixture at the same ratio and fired at different temperatures; the significance was determined by Student’s *t* test (*, *p* < 0.05; **, *p* < 0.01; ***, *p* < 0.005); ns, not significant).

Samples	FS (%)	UUW (γ, g/cm^3^)	WWA/*p* (%)	CS (σ, MPa)	CS-AF (σ_F_, MPa)
FB_0–__900_	1.07 ± 0.04	1.89 ± 0.02	18.00 ± 0.65	15.30 ± 0.78	10.80 ± 0.70
FB_10–__900_	1.43 ± 0.05 ***	1.77 ± 0.01 ***	18.70 ± 0.67 ns	12.70 ± 1.17 ***	9.00 ± 0.56 ns
FB_20–__900_	1.53 ± 0.03 ***	1.70 ± 0.01 ***	19.00 ± 0.66 *	11.90 ± 0.72 ***	8.20 ± 0.46 **
FB_30–__900_	1.66 ± 0.06 ***	1.68 ± 0.02 ***	19.10 ± 0.65 *	10.20 ± 0.39 ***	7.10 ± 0.53 *
FB_40–__900_	1.65 ± 0.05 ***	1.63 ± 0.02 ***	19.50 ± 1.12 ns	8.20 ± 0.32 ***	6.20 ± 0.36 ***
					
FB_0–__1000_	1.52 ± 0.04 ***	1.87 ± 0.02 ns	18.20 ± 0.68 ns	18.00 ± 1.08 *	14.83 ± 0.70 *
FB_10–__1000_	1.53 ± 0.06 ns/ns	1.76 ± 0.01 ***/ns	18.60 ± 0.85 ns/ns	14.20 ± 1.47 */***	9.30 ± 0.46 **/ns
FB_20–__1000_	1.59 ± 0.06 ns/ns	1.72 ± 0.02 ***/***	18.90 ± 0.64 */ns	13.80 ± 0.57 ***/*	10.10 ± 0.90 **/ns
FB_30–__1000_	1.62 ± 0.07 */ns	1.64 ± 0.03 ***/ns	19.00 ± 0.75 ns/ns	13.10 ± 0.41 ***/ns	9.90 ± 0.87 */*
FB_40–__1000_	1.65 ± 0.05 */ns	1.62 ± 0.02 ***/ns	19.10 ± 1.00 ns/ns	11.63 ± 0.51 ***/***	7.80 ± 0.36 ***/*
					
FB_0–__1100_	1.53 ± 0.05 ***	1.85 ± 0.02 ns	17.83 ± 0.57 ns	20.40 ± 1.12 ***	13.90 ± 0.72 ***
FB_10–__1100_	1.58 ± 0.04 ns/*	1.75 ± 0.04 ***/ns	17.83 ± 0.67 ns/ns	21.90 ± 1.15 ns/***	17.80 ± 0.61 */***
FB_20–__1100_	1.62 ± 0.06 ns/*	1.68 ± 0.03 ***/ns	18.00 ± 0.68 ns/ns	22.80 ± 0.68 */***	19.13 ± 0.60 ***/***
FB_30–__1100_	1.64 ± 0.05 */ns	1.62 ± 0.02 ***/*	18.20 ± 0.83 ns/ns	26.20 ± 0.43 ***/***	19.80 ± 0.78 ***/***
FB_40–__1100_	1.68 ± 0.07 */ns	1.60 ± 0.02 ***/ns	18.40 ± 1.13 ns/ns	28.80 ± 0.41 ***/***	24.43 ± 0.47 **/***
					
FB_0–__1150_	1.52 ± 0.07 ***	1.82 ± 0.02 **	16.60 ± 0.68 ns	23.40 ± 1.33 ***	16.50 ± 0.70 **
FB_10–__1150_	1.56 ± 0.05 ns/*	1.66 ± 0.01 ***/***	16.70 ± 0.78 ns/*	26.60 ± 0.59 **/***	19.70 ± 0.62 ***/***
FB_20–__1150_	1.70 ± 0.07 **/***	1.62 ± 0.02 ***/***	17.20 ± 1.16 ns/ns	28.20 ± 0.68 ***/***	23.00 ± 0.44 ***/***
FB_30–__1150_	1.76 ± 0.06 **/*	1.54 ± 0.02 ***/***	18.00 ± 1.16 ns/*	31.50 ± 0.74 ***/***	25.03 ± 1.30 ***/***
FB_40–__1150_	1.80 ± 0.07 ***/*	1.51 ± 0.01 ***/***	117.98 ± 1.12 */*	34.40 ± 0.50 ***/***	29.30 ± 0.98 **/***

## Data Availability

No new data were created or analyzed in this study. Data sharing is not applicable to this article.
